# Health implications of radiological terrorism: Perspectives from Israel

**DOI:** 10.4103/0974-2700.50747

**Published:** 2009

**Authors:** Moti Hagby, Avishay Goldberg, Steven Becker, Dagan Schwartz, Yaron Bar-Dayan

**Affiliations:** IDF Medical Corps, Surgeon General Headquarters, Israel; 1 Faculty of Health Sciences, Ben Gurion University, Beer-Sheva, Israel; 2 School of Environmental Health, University of Alabama, USA; 3 Israeli National EMS (Red Shield of David), Israel; 4 Home Front Command Medical Department, Israel

**Keywords:** Multiple imputation, national trauma data bank, physiological variables

## Abstract

September 11^th^ events taught us, members of the medical community, that we need to prepared for the worst. Nuclear terror is no longer science fiction. Radiological weapons of mass terror come in three flavors: The first one is nuclear. Since 1992, there have been six known cases of highly enriched uranium or plutonium being intercepted by authorities as it passed in or out of the former Soviet Union. Constructing a nuclear fission weapon requires high-level expertise, substantial facilities, and lots of money. All three of which would be difficult, although not impossible, for a terrorist group to pull off without state support. However, terrorists could carry out potential mass destruction without sophisticated weaponry by targeting nuclear facilities using conventional bombs or hijacked aircrafts. Terror attacks could also carry out mass panic and radioactive contamination of people and environment by dispersal of radioactive materials with or without the use of conventional explosive devices. Most medical and para-medical personnel are not familiar with CBRN terror and radiation casualties. To lessen the impact of those potential attacks and provide care for the greatest number of potential survivors, the community as a whole – and the medical community in particular – must acquire the knowledge of the various signs and symptoms of exposure to irradiation and radioactive contamination as well as have a planned response once such an attack has occurred. Based on knowledge of radiation hazards, medical emergency planers should analyze the risks of each scenario, offer feasible solutions and translate them into internationally accepted plans that would be simple to carry out once such an attack took place. The planned response should be questioned and tested by drills. Those drills should check the triage, evacuation routes, decontamination posts, evacuation centers and receiving hospitals. It is crucial that the drill will consist of simulated casualties that will follow the evacuation route from point zero to the ED. Knowledge and exercise will reduce terror (fear) from radiation and help the community as a whole better cope with such an event. This article will review the general information of radiation types, their biological damage, clinical appearance and general concepts of nuclear event planning, focusing on medical response and focus on the Israeli perspective.

## INTRODUCTION

After the World Trade Center collapse, the unthinkable no longer seems impossible.

If a group armed with knives could bring about such devastation, what might they be capable of doing with weapons of mass destruction?[1]

Radiological weapons of mass terror come in three flavors: The first one is nuclear.

Since 1992, there have been six known cases of highly enriched uranium or plutonium being intercepted by authorities as it passed in or out of the former Soviet Union.

Constructing a nuclear fission weapon requires high-level expertise, substantial facilities, and lots of money. All three of which would be difficult, although not impossible, for a terrorist group to pull off without state support.[1]

However, terrorists could carry out potential mass destruction without sophisticated weaponry by targeting nuclear facilities using conventional bombs or hijacked aircrafts. Terror attacks could also carry out mass panic and radioactive contamination of people and environment by dispersal of radioactive materials with or without the use of conventional explosive devices.[2–5]

To lessen the impact of those potential attacks and provide care for the greatest number of potential survivors, the community as a whole – and the medical community in particular – must acquire the knowledge of the various signs and symptoms of exposure to irradiation and radioactive contamination, as well as have a planned response once such an attack has occurred.

This article will review the general information of radiation types, their biological damage, clinical appearance and general concepts of nuclear event planning, focusing on medical response and focus on the Israeli perspective.

## TYPES OF IONIZING RADIATION

Ionizing radiation is a high-energy particle or electromagnetic radiation that deposits energy when it interacts with atoms, resulting in ionization (electron excitation).[6] Regarding biological consequences of ionizing radiation include direct and indirect actions. In the direct mode radiation may directly hit a particularly sensitive atom or molecule in the cell.[6] As a result the cell may either die or malfunction. The radiation can also damage a cell indirectly through the creation of unstable, toxic hyperoxide molecules; which in turn can damage sensitive molecules and afflict subcellular structures.[6] Ionizing radiation can be emitted from atoms of radioactive isotopes, natural (e.g., ^238^uranium) or man-made (^241^americium, ^60^cobalt), or from man-made generators.[6]

### Alpha particles

Alpha particles are massive, charged particles (4 times the mass of a neutron).[6] Because of their size, alpha particles cannot travel far and are fully blocked by the dead layers of skin or by cloths. Alpha particles have negligible external hazards, but when they are emitted within an internalized radionuclide source, they can cause significant cellular damage in the region immediately adjacent to their physical location.[6]

### Beta particles

Beta particles are very light, charged particles that are found primarily in fallout radiation. These particles can travel a short distance in tissue; if large quantities are involved, they can produce damage to the basal striatum of the skin. The lesion produced, a “beta burn,” can appear similar to a thermal burn. In addition, internalized beta emitting isotopes can cause damage similar to alpha emitters.[6]

### Gamma rays

Gamma rays, emitted during a nuclear detonation and in fallout, are uncharged radiation similar to x-rays. They high levels of energy and pass through matter easily. Because of their high penetrability, gamma radiation can result in total body exposure.

Neutrons, like gamma rays, are uncharged, are commonly emitted during the nuclear detonation, and are not a fallout hazard. However, neutrons have significant mass and interact with the nuclei of atoms, severely disrupting atomic structures. Compared to gamma rays, they can cause up to 20 times more tissue damage.[6]

### Gray

Gray (1 joule per kilogram, SI units) is a measure of the absorbed tissue radiation dose. It replaces the former radiation absorbed dose (rad) unit (1 Gray = 100 rad) and is the total amount of energy absorbed per gram of tissue, Gray is used for all forms of ionizing radiation. Different radiation types have different biological effects when absorbed by tissues. To adjust for this difference a Quality Factor (QF)[6] is used.

The dose in gray times the QF yield the *Sievert* (SI units), formerly known as rem (rad equivalent, man). The QF for gamma radiation and x-rays is 1 compared to 20 for alpha particles.[6]

## CLINICAL AND BIOLOGICAL EFFECTS OF IONIZING RADIATION

Cells damaged by high doses of ionizing radiation will suffer irreparable damage that will lead to cell death, usually during the stage of cell replication (apoptosis). Lower doses can inflict damage to DNA molecules that will not lead to cell death, but might cause later effects, including neoplastic transformation.[7]

### Radiation has two types of effects on the organism

#### Deterministic effect

The deterministic effect appears soon after the exposure (hours to months), has a threshold for appearance and its severity is proportional to the radiation dose. The deterministic effect is caused by cell death, mainly in tissues with high cell turnover. Examples are acute radiation sickness and radiation burns.[8–10]

#### Stochastic effect

The stochastic effect appears a long time after the exposure (years), is thought not to have a threshold and its severity is not related to the dose (though the probability of its appearance is). Radiation related malignancy and genetic defects are stochastic effects. The risk for malignancy after radiation exposure is elated to the dose and is added to the general risk for cancer.[8–10]

### Acute radiation sickness

Acute radiation sickness, or syndrome, (ARS) represents the death of cells after exposure to total body ionizing radiation. With no appropriate medical care, the median lethal dose of radiation LD_50/60_ (causing demise of 50% of exposed persons within 60 days) is estimated to be 3.5 Gy. With appropriate medical intensive care, LD_50/60_ will rise to 5–6 Gy.[8–10]

ARS symptoms may vary based on individual radiation sensitivity, type of radiation and the radiation dose absorbed.[6] ARS has four phases: The prodromal phase includes gastrointestinal (GI) symptoms (nausea and vomiting), headache, erythema, elevated core body temperature and malaise. An early onset of symptoms indicates a higher level of exposure. These symptoms can last a few of days.[2–6]

Following the prodromal phase the patient is relatively symptom free. This is the latent phase which can last anywhere from a few days to 6 weeks, – depending on the radiation dose. The higher the dose – the shorter the latent phase.[2–6]

The third and crucial phase is the manifest illness. This phase manifests the clinical symptoms associated with the organ system injured (hematopoietic, gastrointestinal, and cerebrovascular). Table 1 summarizes the pathophysiological appearance at each level of exposure.[2–6]

**Table 1:** Pathophysiological appearance and survival expectancy

Dose range (Gy)	Prodromal effects	Manifest illness effects	Survival expectancy (with no treatment)
0.5–1.0	Mild	Slight decrease in blood cell count	Almost certain
1.0–2.0	Mild to moderate	Early symptoms of bone-marrow damage	Probable (>90%)
2.0–3.5	Moderate	Moderate to severe bone-marrow damage	Possible
3.5–5.5	Severe	Severe bone-marrow damage, slight intestinal damage	Death within 3–6 weeks
5.5–7.5	Severe	Bone-marrow pancytopenia, moderate intestinal damage	Death within 2–3 weeks
7.5–10.0	Severe	Combined gastrointestinal and bone-marrow damage	Death within 1–3 weeks
10.0–20.0	Severe	Gastrointestinal death, neurovascular damage	Death within 5–12 days
>20.0	Severe	Gastrointestinal, neuro- and cardiovascular damage	Death within 2–5 days

Source: Data from reference 7

An early evaluation of the absorbed dose is important in determining the clinical management. One can use clinical dosimetry; assessing the time elapsed from exposure to beginning of prodromal symptoms, or use blood count as dosimetry; the earliest the drop in lymphocyte count, the higher the exposure level.[11] The golden standard for dosimetry is an evaluation of chromosomal aberration, but it demands time and laboratory expertise.[11]

Medical care should include isolation, fluid and electrolytes replacement, anti-emetics, anti-microbial (microbial, viral and fungal) therapy, bone marrow stimulants (in certain circumstances bone marrow transplantation) and psychological support.[11–12]

### Carcinogenesis

Radiation has a stochastic effect leading to carcinogenesis. Based on statistical probability, it is known that exposure dose of less than 1.0 Gy can lead to functional changes that after a latency period of several years may show up as radiation-induced tumors. Absolute risk determination of doses less than 100 mGy is not available and all the risk estimation of carcinogenesis is extrapolated from radiation doses greater than 1 Gy.[6] Some radioisotopes have affinity to specific target organs; radioactive iodine – thyroid, plutonium – bone, and as such, they are presumed to be carcinogenic to those organs, not to other remote organs. Surveys of past human exposures have led to an estimation of added 5%–7% lifetime risk of cancer for every 1 Sievert.[13]

### Psychological effects

An attack involving the release of radiation will likely create uncertainty and fear. Once it is revealed that terrorists have used a radiologic dispersal device, the management of acute psychological and behavioral responses will be as important and challenging as the treatment of radiation-related injuries and illnesses.[12]

Signs and symptoms of autonomic arousal such as tachypnea, tachycardia, nausea, and diarrhea occurring in unexposed patients may be misattributed to the effects of radiation.[12] Psychological distress after a radiologic incident may also manifest as nonspecific somatic complaints. Health care providers who do not have a clear understanding of the risks posed by radiation or the necessary precautions may experience fear and anxiety, resulting in absenteeism, refusal to see patients, and dereliction of duty. Some patients, such as pregnant women, the parents of small children, and children themselves, have special needs and may require additional attention. Patients may also be concerned about the long-term risk of developing cancer, and this concern may persist for years after the event in question. For the vast majority of people, distress and psychological and behavioral symptoms related to the traumatic event exposure will diminish over time. For others, however, symptoms will persist, affect function at home and work, and may result in psychiatric illness.[12,13]

## THE RESPONSE

### Pre-attack planning

Planning the response to nuclear terrorist attack, whether by primitive nuclear device or dispersal of radioactive materials, requires collaboration of several agencies. It must be based on valid assumptions derived from the study of past responses to mass casualty events and drills.[14]

Rational triage of patients and resources is essential so that many can benefit, rather than few.[14] Taking into account that medical personnel or support facilities may be harmed or disabled, (particularly after detonation of nuclear device), the planners should rely on teams that will arrive from outside the immediate damaged circle.[14]

After the exact nature of the attack has been verified, which is simpler in case of detonation or damage to a nuclear facility and more complicated in case of dispersal of radioactive materials, the authorities should be able to setup a mission command.

Since an “outside” aid is usually not available for 24 to 48 h, the local community should be prepared to manage the attack during the early stages; recruit all available skilled rescue personnel, heavy construction equipment and operators. [15–20]

The command should be based on integration of all community and government agencies and should have an organizational structure that will be similar to the command systems in other natural and man-made disasters. It needs to include representatives of all involved disciplines, including a physician skilled in radiation casualties and disaster medicine. [14–20]

In advanced planning, all scenarios need to be taken into account, taking the worst case scenario as a model for planning. After the scenarios are analyzed and the number and severity of potential casualties is determined, solutions should be proposed, based on experience from previous disasters, knowledge and intuition.[14–20]

Professional teams, representing the various disciplines, should process the solutions into plans.

The medical planning team should be based on emergency medical service personnel, who are familiar with the pre-hospital system, and physicians who are familiar with radiation casualties.

Fortunately, most of us have not endured such a disaster. Large-scale drills,[1] which are relatively common among the medical communities, hospitals and army can partially compensate for lack of experience in actual radiological disasters. Such a drill can use simulated casualties that are triaged and evacuated to hospitals or can be a desktop drill that is mentioned to evaluate the plans and the command itself.[2–14]

### The response during the attack

The response is dictated by the nature of the terrorist attack.

#### The RDD scenario

The first scenario, which seems more likely, is the dispersal of radioactive materials.[21] This can be accomplished by using an explosive device in the center of a major city or by simply spraying it from an airplane or other ambulatory spraying device.

Radioactive materials, such as ^60^Co or ^241^Am, are commercially available and not easy to track.

^60^Co is high-energy gamma ray emitter that can cause total body exposure that will lead to ARS. In addition, it predisposes those exposed to future malignant transformations. There is no antidotal treatment for internal contamination and the only available treatment is symptomatic, for ARS.

^60^Co posses an hazard as external and internal contaminator; so protection can be achieved only by shortening the exposure time by physical blocking agents such as lead and by increasing the distance from the radiation source.[21–27]

^241^Am, on the other hand, is an alpha particles emitter, and as so it possesses hazard only as an internal contaminator. The contamination can be caused either by inhalation or via damaged skin. Once it entered the body, it can raise the probability to malignant transformation in the exposed cells. In contrast to ^60^Co, DTPA can be used as an effective treatment to internal contamination with ^241^Am. Face masks can be used as effective protecting devices in the contaminated area.

Table 2 summarizes some of the potential radioactive materials, their daily use, their potential terrorist use and mode of contamination.[22–27]

**Table 2:** Potential contaminating radioactive materials

Agent	Type of irradiation	Daily use	Potential terrorist use	Mode of contamination	Critical body site	Treatment
Americium-241	Alpha	Smoke detectors, research, ground humidity detectors	RDD	Inhalation	Lungs	DTPA
				Skin wounds	Liver bone	
Cesium-137	Beta	Radiotherapy devices	RDD	Lungs	Treated as potassium	Prussian
	Gamma		RED	GI tract wounds	analog	Blue
Cobalt-60	Gamma	Radiotherapy food irradiators	RDD	Lungs wounds	Whole body irradiation	Gastric lavage. No antidot
			RED			
Iodine-131	Beta gamma	Thyroid ablation, reactor cores	RDD	Inhalation GI tract	Thyroid	Potassium-iodide
Strontium- 90	Beta	Former Soviet Union military equipment	RDD	Inhalation	Bone	Lavage oral phosphates
				GI tract wounds		
Iridium- 192	Gamma	Industrial radiography equipment	RDD	Inhalation wounds	Whole body irradiation	None
			RED			

Source: Data from reference 6

#### Classifying the casualties

In radioactive contamination, the injuries are divided into two major groups: the first group of casualties is from “point zero;” they are expected to have trauma injuries if an explosive device is used, to be exposed to high-energy penetrating external radiation (such as ^60^Co) and be contaminated by internal and external radioactive materials. The overall number of casualties if an explosive device was used is expected to be on the scale of dozens.[22–27]

The second group of casualties is from the periphery of the scene; they are expected to be exposed to low-energy penetrating external radiation (such as ^60^Co) and be contaminated by internal and external radioactive materials. The number of casualties from the periphery of the scene is expected to be at the scale of hundreds to thousands.[22–27]

#### The first responders

The first responders to arrive at the site are firefighters, policemen and emergency medical personnel.[2–5]

The most senior person at the scene should take command, surveying the area and carefully assessing the nature of the event and number of victims. In case of dispersal of radioactive contamination by explosive device, it is expected that the nature of the attack will be realized only after the scene will be investigated by RAID (“rapid assessment and initial detection”) teams who will be able to identify the radioactive material.[28] At this phase, a decision should be made whether according to the degree of demolition and number of casualties, the pre-event plan should be activated or that local resources are sufficient.

Once the plan is activated, a command post should be established at the scene and an emergency operations center should be localized outside the danger zone, preferably near or at a communication center.

Since wide areas (depending on weather conditions) are expected to be contaminated they should be declared as restricted areas and unauthorized personnel should not be allowed into it. If many victims are within the potentially contaminated areas, it is wise to include the medical facilities in those restricted areas so that critical patients can be treated without the need to cross the restricted lines and endanger the noncontaminated areas and medical facilities.

#### Triage and evacuation

Trauma 4 casualties, whether contaminated or not, will be evacuated from the immediate scene to the nearest available Trauma Center. These centers should be prepared to triage and decontaminate them, according to their medical status, before entering the building – as in the triage of Chemical Warfare victims.[2–18]

People who are suspected to be contaminated, externally or internally, with no apparent injuries should not be evacuated at this stage to the hospitals. Instead, they should be transported by designated vehicles to an evacuation center; school or large sport arena, where they need to undergo a contamination survey, be decontaminated if necessary, have medical evaluation for ARS and get specific antidote – if indicated.

This evacuation center should be able to receive hundreds to thousands of casualties, have decontamination facilities; showers and dressing rooms, and serve as temporary hostel to those evacuated casualties – until a decision is made whether to hospitalize them for suspected ARS or to send them home after proper registration and medical evaluation.[2–5,18]

As mentioned above, there is a possibility that the radioactive material will be sprayed, rather than used with explosives and the contamination will be discovered only after first casualties will demonstrate ARS or beta burns or even by routine environmental radioactive surveys. In such a scenario, all people in the suspected contaminated area should be called to the evacuation center where they should undergo the same procedure as above; contamination survey, decontamination and medical evaluation.[2–5,18]

#### Radiation protection of the emergency teams

Upon confirmation of a radioactive contamination scenario, all measures should be taken to minimize the number of rescue team members at the immediate scene, to reduce the time of exposure and to supply them with the best protective gear, particularly face masks.[2] At the end of their work, they should be undressed at designated exit posts and transferred to the evacuation center for contamination survey, decontamination and medical evaluation.[2–5,18]

The treatment of the environment, which is a complex federal task, will not be discussed in this review.

#### Attack on a nuclear facility

The second scenario is that of an intentional crash of a large commercial airliner into a nuclear facility. Although nuclear power plans were not designed to stand such a crash, it will not cause nuclear explosion. It can rather cause a damage to the outer layer of the facility or even rupture the reactor core, which in turn can cause release of fusion product found in reactor fuel rods.

Post-destruction winds will determine the fallout pattern. Most of the fallout in this case will be of radioactive Iodine-131, 132, 134, and 135 (NRC News, September 21, 2001).

All nuclear facilities should have emergency response plans to enable the mitigation of impact in the event of such a release; supply of Potassium-Iodide tablets for the public in the immidiate vacinity, evacuation plans for the employees and the general public and contamination survey posts for those evacuated from the contaminated area. Those plans, in general, should be the same as for the above scenario.[22–27]

#### Nuclear detonation

The third and most horrifying scenario is that of nuclear detonation in which the scene will be seen as the one that Dr. Sasaki, of Hiroshima, confronted after the detonation on August 6, 1945; “The people…wept and cried, for Dr Sasaki to hear, ‘sensai! Doctor!’…Bewildered by the numbers, staggered by so much raw flesh, Dr. Sasaki lost all sense of profession and stopped working as a skillful surgeon and a sympathetic man; he became an automaton, mechanically wiping, daubing, winding.”[29] Taking into account that the city's medical facilities almost entirely destroyed, effective care was virtually impossible.[30]

Nowadays, in western cities, not all medical facilities will be destroyed, but certainly will be damaged by a nuclear detonation. It is possible, although very expensive, to prepare for such scenario but is beyond the scope of this review. It is also noteworthy that most western countries during the Cold War preferred to invest all they could in preventing such a scenario instead of making plans of how to deal its results. Intelligence agencies all over the world are trying to do the same in the post Cold War era; tracking and making efforts to prevent leakage of weapons with usable nuclear materials from the Former Soviet Union.

#### Triage after nuclear detonation [Figure 1]

Triage (similar to other disaster scenarios) needs to be quick and effective and should be performed by the senior medical officer on scene or in the Emergency Department (ED).[11–14] If there is an injury necessitating immediate hospital treatment or whether only radioactive contamination is involved, in which case the victim needs to be triaged to the evacuation center.[2]

**Figure 1:**
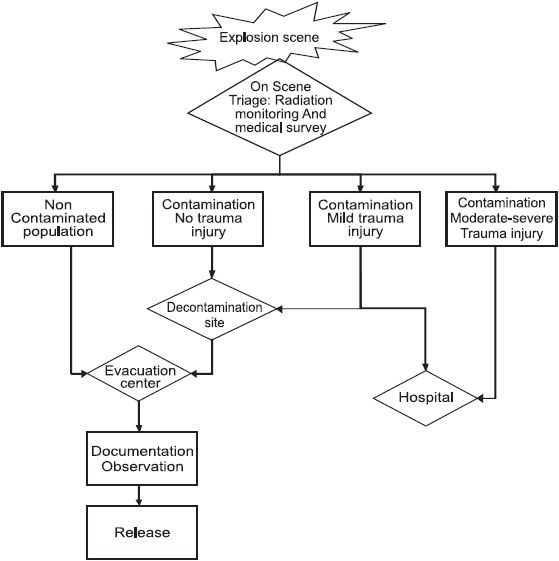
The figure illustrates the casualties evacuation scheme from the scene

Correct triage can reduce patient load in the ED from casualties not needing hospitalization and will allow medical attention to focus on casualties that might die without appropriate trauma care.[19]

The ED and hospitals should be familiar with contingency plans and the principles of care for radiation injuries. It is advisable that in every major city one hospital will be designated for such scenarios.[2–5] These hospitals need to have their own plans for such an event, have assigned areas for contaminated casualties with appropriate decontamination facilities and protective gear for the medical and nonmedical personnel.[2–22] Equipment for contamination survey and trained personnel to operate it are necessary.[18] Periodical exercises and drills are necessary for hospital teams and for better coordination with local and governmental agencies and the local rescue personnel.[18]

Since a significant portion of the damage caused by radiation will not be evident for months, or even years, it is mandatory that all potential casualties will be medically evaluated and documented any long-term follow up instituted.

Records should include demographic data, the exact place of the victim at the moment of the event, contamination survey results if available, treatment if given, signs and symptoms pointing to ARS and blood count if performed. The records should be kept for future medical follow up both in case of ARS that can develop in the consecutive days or for cancer screening programs in the years to come.[16,18]

## THE ISRAELI PERSPECTIVE

Hoffman *et al*. emphasized the need for a national preparedness program for such mass casualty events, led by national health systems.[31] Periodic drills for unconventional terrorism and radiological terrorism are conduced in Israel since 1997.[32] Israel has heightened security at airports, seaports, and border sites to prevent radiological dirty bombs from entering the country. New measures include scanning freight cargo at seaports. Israel has also increased security at facilities that could be sources of material for dirty bombs.[33]

In the ministry of defense, they are preparing to different types of terrorism. Among them, they have recently conducted a large scale top table drill for the managers of the emergency care systems in Israel. The drill simulated a terrorist event that include the use of radiological materials and is part of a long term periodic drills to all of the components of the Israeli emergency services in all the levels and in different scales that are aimed to improve the Israeli preparedness for a wide spectrum of possible scenarios.[34] According to a governmental decision in Israel, the minister of defense has the overall responsibility for radiological terrorism, which includes nonconventional elements. In the drill, a spread of radiological materials by a dirty bomb was discussed. The police, Israeli defense forces, home front command, Israeli emergency medical services, fire brigades, ministry of environment, ministry of health and other organizations took part in such an event, and their representatives took part in this drill with full collaboration between all the organizations.[34]

## CONCLUSIONS

Nuclear terror, presenting in three possible scenarios, is no longer science fiction. September 11^th^ events taught us, members of the medical community, that we need to prepared for the worst.

Most medical and para-medical personnel are not familiar with CBRN terror and radiation casualties. Management of these types of incidents should be taught and the personnel and relevant agencies need to be trained and drilled.

Based on knowledge of radiation hazards, medical emergency planers should analyze the risks of each scenario, offer feasible solutions and translate them into internationally accepted plans that would be simple to carry out once such an attack took place.

The planned response should be questioned and tested by drills. Those drills should check the triage, evacuation routes, decontamination posts, evacuation centers and receiving hospitals. It is crucial that the drill will consist of simulated casualties that will follow the evacuation route from point zero to the ED.

Knowledge and exercise will reduce terror (fear) from radiation and help the community as a whole better cope with such an event.
